# Testicular Vascularization after Pediatric Inguinal Hernia Repair: A Systematic Review and Meta-Analysis

**DOI:** 10.3390/children11040409

**Published:** 2024-03-29

**Authors:** Roxanne Eurlings, Rianne E. M. Killaars, Ruben G. J. Visschers, Wim G. van Gemert

**Affiliations:** 1 Research Institute of Nutrition and Translational Research in Metabolism (NUTRIM), Faculty of Health, Medicine and Life Sciences (FHM), Maastricht University, Universiteitssingel 40, 6229 ER Maastricht, The Netherlands; 2 Department of Pediatric Surgery, MosaKids Children’s Hospital, Maastricht University Medical Center+, P. Debyelaan 25, 6202 AZ Maastricht, The Netherlands; 3 European Consortium of Pediatric Surgery (Maastricht University Medical Center+, Uniklinik Aachen, Centre Hospitalier Chrétien Liège), P. Debyelaan 25, 6229 HX Maastricht, The Netherlands

**Keywords:** pediatric inguinal hernia, testicular vascularization, resistive index, laparoscopic inguinal hernia repair, open inguinal hernia repair

## Abstract

Background: The effect of pediatric inguinal hernia repair (IHR) on testicular vascularization remains unclear. Manipulating the spermatic cord during surgery may reduce blood flow due to edema and vasoconstriction. This can lead to testicular atrophy. The study aims to review current knowledge of testicular vascular impairment following IHR in children. Methods: A systematic literature search was conducted in PubMed/Medline, Embase, Cochrane Library, and Web of Science. Methodological quality was assessed using validated tools. Data were extracted, and a pooled data analysis was performed. Results: Ten studies were included in the systematic review. Six of these studies were eligible for meta-analysis. This revealed a significant decrease in testicular vascularization during the short-term follow-up (1 day–1 week) after IHR using the open surgical approach. This decrease was not present after laparoscopic intervention. There was no more increased resistance in the vessels at long-term follow-up (1 month–6 months), suggesting that the impaired vascularity is only temporary. Conclusions: There seems to be a short-term transient vascular impairment of the testis after open IHR in children. This might be of clinical relevance to prefer the laparoscopic approach for IHR in children, even though the open approach is the gold standard, in contrast to adult IHR. The impact on testicular function and sperm quality later in life remains unclear. Comparative studies of both techniques are needed to determine if there is a significant difference in testicular vascularity. Long-term studies are necessary to assess the impact of transiently reduced vascularity on sperm quality and fertility later in life.

## 1. Introduction

Inguinal hernia is a common problem in the pediatric population, making inguinal hernia repair (IHR) the most frequently performed procedure in pediatric surgery [1]. The procedure is safe and effective, with a low complication rate [2,3,4]. Testicular atrophy is a potential major complication that can occur following both open and laparoscopic IHR. The reported incidence varies based on different factors. Older literature describes testicular atrophy rates between 0% and 5% after uncomplicated IHR in children and between 2.3% and 15% for IHR after incarceration [5,6,7,8,9,10,11,12]. In more recent literature, the reported incidence seems to have decreased to rates as low as 0.028–0.3% after uncomplicated IHR, both with the open and laparoscopic approach [13,14,15]. Especially in younger children (<2 years), the risk of ischemia of the testis after surgery is increased due to the lack of collateral blood flow [7,11,13,16,17]. Another possible risk factor is an undescended testicle [13,18].

There seems to be an ongoing debate about the impact of IHR in children on testicular vascularization and possible subsequent testicular atrophy. It is also unclear what the role of the surgical technique is on the vascularity of the testis. The manipulation of the funicular structures is almost non-existent in laparoscopic repair compared to open repair. This could serve as an argument for preferring a laparoscopic approach over an open approach, which is still the accepted standard in IHR in children, in contrast to the adult IHR.

Several prospective studies have been conducted adressing the topic of testicular vascularization following pediatric IHR. So far, no systematic review with a meta-analysis has been performed to summarize these studies. Therefore, the aim of this paper is to review and compare the existing literature on testicular vascularization before and after both laparoscopic and open uncomplicated IHR in the pediatric population.

## 2. Materials and Methods

This systematic review and meta-analysis was conducted according to the Preferred Reporting Items for Systematic Reviews and Meta-analyses (PRISMA) statement [19,20]. The protocol was registered in PROSPERO (CRD42023445128).

### 2.1. Literature Search

The initial search was performed on 17 July 2023 in the PubMed/Medline, Embase, Cochrane Library, and Web of Science databases. The search was last repeated on 30 October 2023 to check for the most recent updates in the literature. The following search string, combining MeSH terms and/or text words based on the PICOTS, was used: (((“Hernia, Inguinal” [Mesh]) OR (inguinal hernia)) AND ((“Infant” [Mesh]) OR (“Child” [Mesh]) OR (“Adolescent” [Mesh]) OR (children) OR (pediatric) OR (pediatric))) AND ((testicular vascularization) OR (testicular artery)). No search terms for the intervention were included to ensure all possible interventions (laparoscopy or conventional open surgery) were included in the resulting articles. No restrictions regarding the date of publication or publication type were applied.

### 2.2. Eligibility Criteria

Inclusion and exclusion criteria were defined using our predefined PICOTS. All participants must be under 18 years of age and have undergone surgery, either through laparoscopy or an open surgical approach, for uncomplicated inguinal hernia. Emergency procedures (e.g., following incarceration) were excluded. The primary outcome measure was the resistive index (RI), calculated by comparison of the peak systolic velocity (PSV) and the end-diastolic velocity (EDV) in the vessels ((PSV − EDV)/PSV). This measure gives an indication of the resistance in the vascular bed that is supplied by the vessels where the PSV and EDV are measured with Doppler ultrasound (DUS). It is a widely accepted measure to quantify testicular vascularization in pediatric patients. Testicular volume and other outcomes regarding testicular vascularity were secondary outcome measures. No exclusion criteria were formed based on the number of participants in the study or the length of follow-up. All case series, prospective cohorts, and comparative studies were eligible for inclusion.

### 2.3. Article Selection

After the removal of duplicate records, all articles retrieved by the search as described above were screened based on title and abstract. The potentially eligible studies were then screened based on the full text. Two reviewers (RE and RK) independently conducted the screening process. Inconsistencies were resolved by discussion and/or consultation with a third reviewer (RV).

### 2.4. Data Extraction and Statistical Analysis

All relevant data, including study characteristics and outcomes, were collected in a predesigned database. Consecutively, statistical analysis was performed using IBM SPSS Statistics for Windows, version 28.0 (IBM Corp., Armonk, NY, USA). Heterogeneity was quantified using a chi-squared test. The data of all studies reporting RI of the centripetal artery preoperatively with a comparison to the RI postoperatively were pooled using the random-effects model in case of a high level of heterogeneity (I^2^ > 50%). If I^2^ was less than 50%, pooling was conducted according to the fixed-effects model [21]. A weighted mean difference, along with the corresponding 95% confidence interval (CI), was calculated for short-term follow-up (1 day–1 week) and long-term follow-up (1 month–6 months) per surgical technique (laparoscopy or open). A *p*-value of less than 0.05 was considered to indicate statistical significance. Lastly, a funnel plot was made to assess possible publication bias.

### 2.5. Risk of Bias Assessment

The methodological quality of the eligible non-comparative studies was evaluated using the Methodological Index for Non-Randomized Studies (MINORS) tool [22]. Eight items were scored: 0 (not reported), 1 (reported but inadequate), or 2 (reported and adequate). The overall scores were calculated as a sum of the sub-scores and categorized as very high risk of bias (0–4), high risk of bias (5–8), moderate risk of bias (9–12), and low risk of bias (13–16). 

For non-randomized comparative studies, the Risk of Bias in Non-randomized Studies of Interventions (ROBINS-I) tool was used [23,24]. Seven different items were scored as being ‘low’, ‘moderate’, ‘serious’, or ‘critical’ risk of bias if anything on that subject was reported in the paper. The different scores on the subdomains were then combined into an overall judgment. Two independent reviewers (RE and RK) performed the risk of bias analysis. In case of discrepancies, the matter was discussed, and a third reviewer (RV) was consulted until a consensus was reached.

## 3. Results

### 3.1. Study Selection

**Figure 1:**
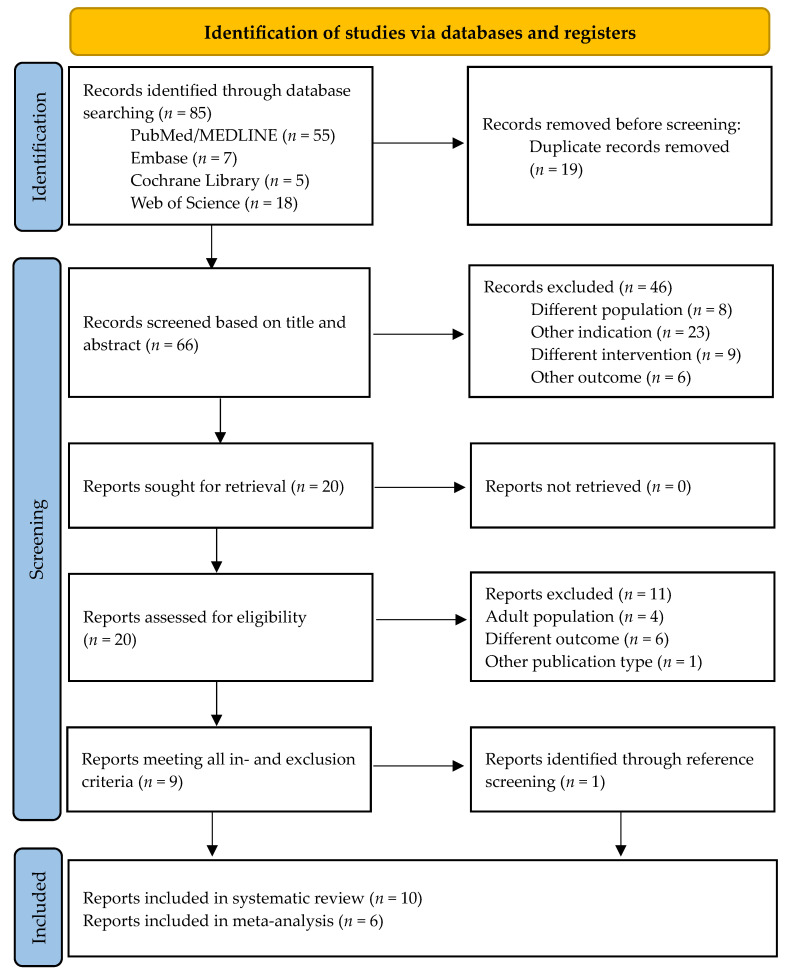
Flow chart of the study selection process [35].

The initial search yielded 85 hits. After removing duplicates, 66 records were screened based on title and abstract. A total of 46 articles were excluded because they did not meet the inclusion criteria. Through a full-text assessment of the remaining articles, nine records were identified that were eligible to be included in the systematic review [25,26,27,28,29,30,31,32,33]. The references of these nine papers were screened, and one additional paper was identified as meeting all inclusion criteria [34]. This led to a total number of ten studies to be included in the systematic review. Of these ten records, six studies reported preoperative and postoperative resistive index of the testicular arteries. These were included in the meta-analysis. During the final search, one additional recent record was found through PubMed. This report did not meet the inclusion criteria. The selection process is summarized in Figure 1.

### 3.2. Study Characteristics

All of the included studies were prospective cohort studies, and no randomized controlled trials were found. Two of the included records reported outcomes for both laparoscopy and conventional open surgical approach [28,30], three studies reported the measurements of testicular vascularization after laparoscopic IHR [26,29,33], and five studies described the results after open surgery [25,27,31,32,34]. Of the comparative and non-comparative studies describing laparoscopy, two reported using the percutaneous internal ring suturing (PIRS) [29,30]; in one study, they performed the laparoscopic repair by purse string-suture [26], and in one, they used both purse string-suture and N-suture [28]. The N-suture and purse string-suture are both trans-peritoneal intra-corporal approaches, making use of three laparoscopic ports. The PIRS technique is a pre-peritoneal approach, making use of only one laparoscopic port for the endoscope. The ligature is made via an extra-corporeal approach with the help of a long needle (either an 18-gauge injection needle or an epidural needle) with a suture inside the barrel of the needle. One record describing a laparoscopic repair did not disclose which suturing technique was used [33]. All the studies compared postoperative values to preoperative measurements. Most studies reported short-term (1 day to 1 week) and long-term (1 month to 6 months) follow-up, with one study having a maximum follow-up of 123 months [34]. The mean age ranged from 20 days to 14 years. All study characteristics are summarized in Table 1.

### 3.3. Methodological Quality

**Figure 2:**
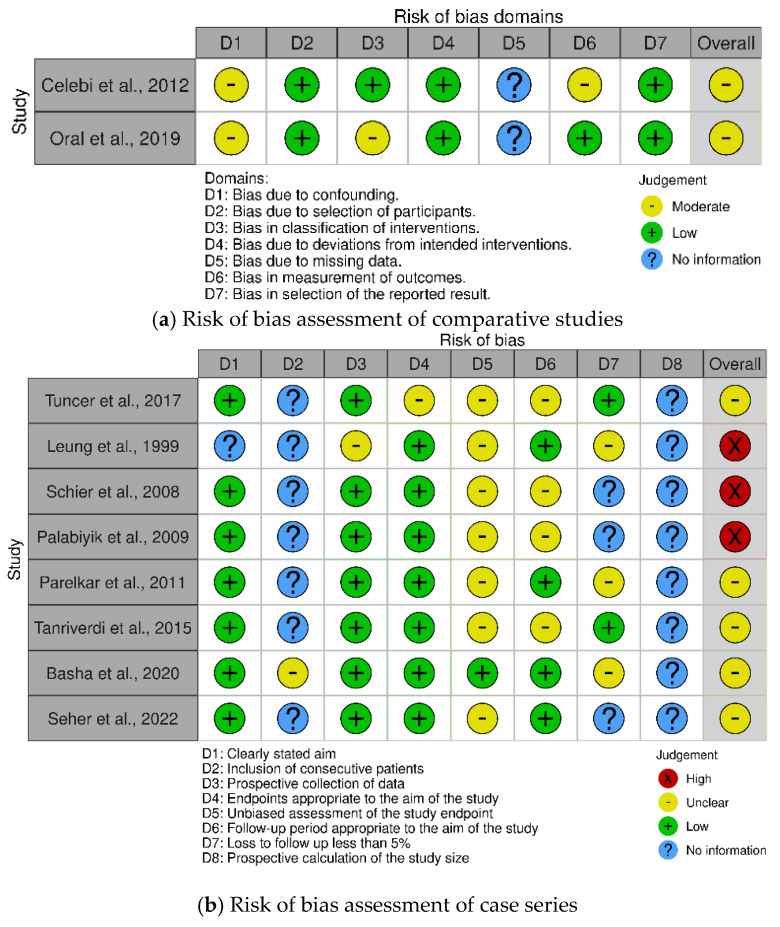
Summary of the risk of bias of the included articles in the systematic review. (**a**) The risk of bias in the studies comparing testicular vascularization after laparoscopic inguinal hernia repair vs. open surgery was assessed with the ROBINS−I tool [28,30]. (**b**) The risk of bias in the studies describing testicular vascularization after laparoscopic inguinal hernia repair or open surgery was assessed with the MINORS tool. Summary plots were created with the robvis tool [25,26,27,29,31,32,33,34,36].

Risk of bias assessment with the ROBINS-I tool for the two comparative studies showed that both studies have a moderate risk of bias [28,30]. This was mainly due to the fact that there was no correction for confounding, it was not mentioned if the radiologist measuring the outcomes was blinded, and there was no mention of missing data (e.g., if it was possible to calculate the RI for every participant, if all participants showed up for follow-up). The summary of the risk of bias assessment for the comparative studies can be found in Figure 2a.

Regarding the non-comparative studies reporting the vascularization of the testis after either laparoscopic or open IHR, three studies have a high risk of bias, and the rest have a moderate risk of bias. This is mainly because almost none of the studies described in consecutive patients were asked to participate in their study, possibly leading to selection bias. Furthermore, most studies did not blind the radiologist, and some of them did not report if one or more radiologists performed the radiologic assessment. This would mean there might be a chance of high inter-observer variability. None of the studies reported performing a prospective power calculation to estimate the necessary sample size. A summary of the risk of bias in the non-comparative studies can be found in Figure 2b.

The risk of publication bias across the six studies included in the meta-analysis was assessed through the creation of a funnel plot for the outcome RI at long-term follow-up for all surgical techniques. The overall *p*-value = 0.944 for the effect size estimates according to Hedges’g and visual assessment confirmed that there seems to be no evident asymmetry in the funnel plot. This means that major publication bias is not probable; however, given the small amount of data included, a sound conclusion about possible publication bias cannot be assumed.

### 3.4. Primary Outcome

#### 3.4.1. Preoperative Resistive Index Compared to Postoperative Resistive Index

Six of the included studies reported the preoperative and postoperative RI of the centripetal artery [25,26,27,28,29,30]. An overview of the values is given in Table 2. All studies compared the preoperative values to the postoperative values, either at short-term follow-up (1 day–1 week) or at long-term follow-up (1 month–6 months). Only Palabiyik et al. (2009) and Basha et al. (2020) reported a statistically significant difference when comparing the preoperative RI to the early follow-up values (*p*-value = 0.0008 and <0.0001, respectively) [25,27]. These are the two studies that only report RI after an open surgical approach. Palabiyik et al. (2009) also found statistical significance between early and late follow-up (*p*-value = 0.036) [25]. Çelebi et al. (2012) found a slight increase in RI at early follow-up for the open technique group, which was not seen in the laparoscopy group. However, the increase was not statistically significant [28]. None of the studies reported a statistically significant difference when comparing the preoperative values of the RI with the late follow-up.

**Figure 3:**
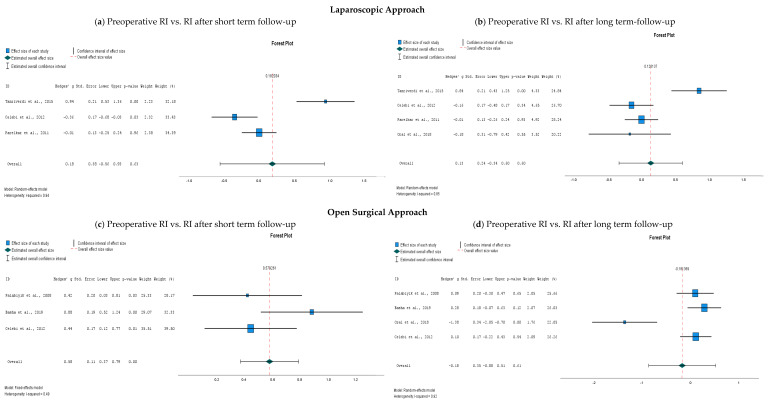
Forest plots of a comparison of the resistive index (RI) of the centripetal artery of the testis after inguinal hernia repair; (**a**) preoperative RI compared to short-term follow-up (1 day–1 week) after laparoscopy [26,28,29]; (**b**) preoperative RI compared to long-term follow-up (1 month−6 months) after laparoscopy [26,28,29,30]; (**c**) preoperative RI compared to short-term follow-up (1 day–1 week) after an open surgical approach [27,28,29]; (**d**) preoperative RI compared to long-term follow-up (1 month–6 months) after an open surgical approach [27,28,29,30]. Abbreviations: RI, resistive index; Std., standard deviation. The reported upper and lower limits are at the 95% confidence interval.

The data (RI for the centripetal artery, preoperative values compared to postoperative values) were pooled according to surgical technique and according to length of follow-up (Figure 3). Only for the data pooling of the RI of the short-term follow-up for the open surgical approach, the fixed effects model was used since the heterogeneity was low (I^2^ < 0.5); for the other analyses, the random effects model was used as I^2^ was > 0.5. I^2^ is reported with each forest plot in Figure 3. The data pooling revealed a statistically significant difference in preoperative RI and RI after short-term follow-up after the open surgical approach (*p* = 0.000, see Figure 3c). The differences between preoperative RI and RI at both short-term and long-term follow-up after laparoscopy were not significantly different (*p* = 0.63 and 0.60, respectively, see Figure 3a,b). In addition, there was no significant difference between the open approach and the long-term follow-up (*p* = 0.61, see Figure 3d).

#### 3.4.2. Resistive Index Values after Laparoscopy Compared to Open Surgery

Two studies reported measurements of RI for both laparoscopic IHR and the open approach [28,30]. Çelebi et al. (2012) did not report a statistical comparison between the different surgical approaches, only between pre-and postoperative RI values [28]. We performed the comparison of means (with an independent samples *t*-test). No statistically significant differences were found between laparoscopic surgery and open surgery at either time point of the outcome measurement. *p*-values are represented in Table 3.

These findings are in line with what is reported by Oral et al. (2019) [30]. They also described no significant difference when comparing RI values before and after both surgical techniques (*p* = 0.727 and 0.220 respectively) [30].

### 3.5. Secondary Outcomes

#### 3.5.1. Testicular Volume

Four studies reported outcomes regarding testicular volume [26,27,31,34]. Data pooling was not possible since the parameters were not the same in all the studies; some reported the absolute testicular volume, while others calculated the ratio of the testicular volume of the operated side compared to the not-operated side. The results of the parameters reflecting testicular volume are represented in Table 4. Note that the findings of Tuncer et al. (2017) were excluded from this summary because they did not report separate data for their included pathologies, and this report only focused on IHR [31]. No study reported statistically significant differences in testicular volume when comparing preoperative measurements with postoperative measurements.

#### 3.5.2. Other Clinical Outcomes

None of the articles reported any cases of testicular atrophy.

Seher et al. (2022) used the vascular index (VI) as a measurement to quantify testicular vascularization before and after opening IHR in children [32]. The VI is the ratio of the Doppler signal received pixels in the region of interest that is drawn on the tissue, compared to all the pixels in the region of interest. They reported significantly different VI values preoperatively compared to postoperatively, with the VI being significantly lower before the surgery (*p* < 0.000). Postoperatively, the testicular vascularization increased and restored to values resembling the values of the contralateral healthy side. However, no significant difference was observed between both pre- and postoperative measures compared to healthy contralateral testicular vascularization; no *p*-value was reported for this analysis in the article.

Schier et al. (2008) made use of a novel technique, O2C (“oxygen to see”), to monitor testicular vascularization after IHR [33]. This neuromonitoring device makes use of a combination of light spectroscopy and laser Doppler techniques. All the measurements were performed before and after anesthesia, before and after surgery (while the patient was under anesthesia), and at 6 weeks postoperatively. They did not find any differences in either of the pre-anesthesia measurements and at follow-up compared to those of matched controls. However, test statistics, confidence intervals, or *p*-values are not reported in the article.

## 4. Discussion

The current study’s aim was to investigate potential alterations of testicular vascularization after IHR in children. Procedures performed with both a laparoscopic approach and a conventional open approach were included. No significant changes in testicular vascularization were observed at short-term and long-term follow-up after the laparoscopic procedure. However, these results must be interpreted with discretion since the heterogeneity was considerable (I^2^ > 50%). The meta-analysis revealed a statistically significant difference in testicular vascularization at short-term follow-up after open IHR compared to the preoperative values. The results at long-term follow-up were not significantly different, suggesting that the decreased vascularity might be transient and normalize after a certain amount of time. Seher et al. also measured the vascularity of the healthy contralateral testicle. They did not find a significant difference between preoperative and postoperative measures [32]. Parelkar et al. calculated the ratio of the testicular volume of the herniated side and the contralateral healthy side. They also did not find a significant difference between preoperative and postoperative measures [26]. These findings indicate that contralateral vascularity has probably no repercussions. The pathophysiology of this phenomenon is thought to originate in the manipulation of the testicular vascular structures during surgery, which leads to vasoconstrictions and edema [26,27,28,37]. This results in increased arterial resistance in the testicular vessels and may potentially lead to testicular ischemia [25,26,27,28,37,38,39]. There is considerably more manipulation of the spermatic cord while performing an open IHR as compared to a laparoscopic approach, which explains why there is a significant increase in vascular resistance at short-term follow-up after the open procedure and not after the laparoscopic surgery. In addition, there might be more manipulation of the spermatic cord depending on the experience of the operation surgeon. Once the edema is decreased or resolved, the vascular resistance normalizes, and subsequently, there is no significant difference in RI in our study anymore. For some, this might be an additive argument for preferring laparoscopic IHR in children. In contrast to the adult IHR, which is standardly a laparoscopic procedure, the accepted standard in children is still an open approach for IHR.

None of the studies reported any cases of testicular atrophy. Since the postoperative vascular alteration is only temporary, it might be assumed that this does not lead to testicular atrophy or hypotrophy. However, the maximum length of follow-up of the included studies was between 1 and 6 months, except for Leung et al., who had a maximum follow-up of 123 months [25,26,27,28,29,30,31,32,33,34]. Sonderman et al. found in a large cohort of nearly 9000 children undergoing IHR that 30% of the testicular atrophy cases were diagnosed within the first year after surgery and 75% within 3 years [13]. A longer follow-up is necessary to determine the correlation between the transient altered vascularity of the testis after open IHR and testicular atrophy/ hypotrophy. Alternatively, the question can be raised if ischemic preconditioning plays a role in protecting the testis after a period of decreased vascularity, similar to the protective effect this has on the testis after torsion-detorsion injury [40,41,42,43,44,45].

Furthermore, it is unclear if this decreased vascularity, even though it spontaneously normalized after some time, potentially has an impact on future testicular function. Literature on this subject is very limited. Pinggera et al. report that in adults, an RI > 0.6 is significantly correlated with lower sperm counts (*p* < 0.001) [46]. It is unclear how long this increased resistance has to be present to lower the quality of the sperm. An experimental study in bulls showed that spermatogenesis was decreased or even totally absent after restriction of the arterial testicular blood flow for 1.5 years [47]. In the studies in this report, the increased vascular resistance decreased to normal values after a couple of months. However, it might be possible that even this short-term decreased vascularity might lead to subfertility later in life. In a large cohort study by Yavetz et al., they found that 6.65% of men attending a fertility clinic had an IHR in their medical history, compared to a 5% prevalence of IHR in the standard population at the time of the study [48]. They also found that the semen quality, measured as sperm concentration, motility, and morphology, was reduced compared to these parameters in fertile men [48]. However, a prospective comparative study by Silber et al. did not find a correlation between a history of pediatric IHR and the quality of sperm later in life [49]. Zendejas et al. reported an infertility rate of 4.7% in an American retrospective cohort study of 213 male and female patients undergoing IHR in childhood, which is even lower than the prevalence of 9–13% in the general population in the United States [50].

In addition, even though the meta-analysis showed a significant difference in resistance in the centripetal artery at short-term follow-up after open surgery, this review did not reveal any significant differences in testicular vascularization when comparing laparoscopy and the open surgical approach for the pediatric IHR. However, a meta-analysis of this comparison was not possible since only two studies reported outcomes of both techniques and one of those did not perform the comparison themselves. In addition, the studies reported different methods of laparoscopic technique; Çelebi et al. used the N-suture and the purse string suture, while Oral et al. reported the outcomes for the PIRS technique [28,30].

### Limitations

There are several limitations to this review and meta-analysis, most of them due to considerable heterogeneity. Although the age range of all studies mostly overlapped, some of the studies excluded younger children, which is an age group that is the most at risk for vascular complications from this surgery due to lacking collateral blood flow. Furthermore, this heterogeneity also manifests in the inclusion criteria of the different studies. Some reports excluded patients undergoing emergency IHR, while others did not report if they enforced this exclusion criterion. In addition, as already mentioned, there are differences in the laparoscopic technique used for hernia repair. All these parameters mentioned above could lead to confounding in the pooled data analysis. Unfortunately, meta-regression analysis to assess this possible confounding could not be performed because of the small number of studies included in the meta-analysis. Lastly, most studies included had a moderate risk of bias, and three studies had a high risk of bias. Even though no studies had extremely poor quality and most of the higher risk of bias was due to lack of reporting of certain information, the moderate and high risk of bias of all studies combined still negatively impacts the solidness of the conclusion that can be drawn from this review. Lastly, the major limitation is that only six of the included studies reported the primary outcome measure and that within these six records, different techniques of surgery were reported, meaning that, per analysis, only a maximum of four studies could be compared.

It is clear that more research is necessary, preferably with a long-term follow-up, to address testicular development and function (subfertility/infertility) later in the life of the patient. In this way, a possible correlation between the temporarily decreased testicular vascularization and sperm quality can be determined in the future. In addition, more comparative studies need to be conducted to test for superiority or non-inferiority of one of the surgical techniques when it comes to testicular vascularization since we only found a significant difference in vascularity at short-term follow-up after the open surgical procedure for IHR.

## 5. Conclusions

In conclusion, shortly after opening IHR in children, testicular vascularization is significantly decreased. This decrease was not observed after laparoscopy, meaning that this approach is possibly preferable regarding the impact on testicular vascularization. This can have relevant clinical implications since the gold standard for IHR in children is still an open procedure, in contrast to the adult IHR. However, the vascularity normalized a couple of months after surgery, and the significant difference was no longer observed. It is unclear if this transient decrease in vascular flow has an impact on testicular volume and function and, thus, perhaps on sperm quality later in life. More comparative studies between both techniques need to be performed to investigate if there is a significant difference when regarding testicular vascularity. Long-term studies are necessary to assess the impact of transiently reduced vascularity on sperm quality and fertility later in life.

## Figures and Tables

**Table 1:** Study characteristics of the included studies describing testicular vascularization after inguinal hernia repair in children.

Author and Publication Year	Country	Study Design	Level of Evidence	Indication	Operation Technique	Intervention Comparison	No. of Participants	No. of IH	Mean Age (Range)	Duration of FU in Months	Outcome Measurement	Reported Outcomes
Tanriverdi et al., 2015 [29]	Turkey	Prospective study	VI	Unilateral IH	Laparoscopic repair (PIRS)	Preoperative measures	49	49	5.27 y (1 y–14 y)	1	DUS	PSV, EDV, RI
Palabiyik et al., 2009 [25]	Turkey	Prospective study	VI	Unilateral IH	Open approach (High ligation)	Preoperative measures	51	51	6.33 y (2 y–14 y)	1	power DUS	PSV, EDV, RI
Çelebi et al., 2012 [28]	Turkey	Prospective study	VI	Unilateral IH	Laparoscopic repair (N-suture and Purse string)	Open approach Contralateral side Preoperative measures	72	72	5.65 y	1	DUS	PSV, RI
Basha et al., 2020 [27]	Egypt	Prospective study	VI	Unilateral and Bilateral IH	Open approach (High ligation)	Preoperative measures	60	63	9.46 m ± 14.46 m (2 m–6 y)	6	color DUS	PSV, EDV, RI Testicular volume
Parelkar et al., 2011 [26]	India	Prospective study	VI	Unilateral and Bilateral IH	Laparoscopic repair (Purse string)	Contralateral side Preoperative measures	100	125	4.45 y (1.5 m–12 y)	6	color DUS	PSV, EDV, RI Testicular volume
Oral et al., 2019 [30]	Turkey	Prospective study	VI	Unilateral IH	Laparoscopic repair (PIRS)	Open approach Contralateral side Preoperative measures	40	40	6.80 y ± 3.5 y	1	SMI (color DUS)	RI
Tuncer et al., 2017 [31]	Turkey	Prospective study	VI	Inguinal hernia Hydrocele Cord cyst	Open approach (High ligation)	Preoperative measures	23	11	3.59 y ± 2.67 y (20 d–12 y)	1	color DUS	PSV Testicular volume
Leung et al., 1999 [34]	Hong Kong	Prospective study	VI	Unilateral IH	Open approach (High ligation)	Preoperative measures	173	173	(10 m–179 m)	123	US	Testicular volume
Seher et al., 2022 [32]	Turkey	Prospective study	VI	Unilateral IH	Open approach (High ligation)	Contralateral side Preoperative measures	44	44	3.8 y (0 y–10 y)	6	SMI (color DUS) + SWE	Testicular volume VI Tissue elasticity/stiffness
Schier et al., 2008 [33]	Germany	Prospective study	VI	Unilateral and Bilateral IH	Laparoscopic repair (No technique reported)	Matched controls Preoperative measures	65	78	(6 w–11 y)	1.5	O2C	O2 Saturation at venous end of capillaries Hb in micro vessels Flow, Velocity

Abbreviation: IH, inguinal hernia; FU, follow-up; PIRS, percutaneous internal ring suturing; y, year; m, month; w, week; d, day; DUS, Doppler ultrasound; PSV, peak systolic velocity; EDV, end-diastolic velocity; RI, resistive index; SMI, superb microvascular imaging; SWE, shear wave elastography; VI, vascular index; O2C, “oxygen to see”, a neuromonitoring device combining light spectroscopy and laser DUS.

**Table 2:** Resistive index of the centripetal artery before and after inguinal hernia repair in the respective study populations.

Study	Technique	Preop RI (Mean ± SD)	Early Postop FU RI (Mean ± SD)	Late Postop FU RI (Mean ± SD)	Preop vs. Early FU (p-Value)	Preop vs. Late FU (p-Value)	Early vs. Late FU (p-Value)
Tanriverdi et al., 2015 [29]	Laparoscopic repair (PIRS)	0.59 ± 0.04	0.62 ± 0.02	0.62 ± 0.03	0.21	0.28	0.856
Parelkar et al., 2011 [26]	Laparoscopic repair (Purse string)	0.499 ± 0.36	0.497 ± 0.35	0.495 ± 0.33	0.296	0.122	0.684
Çelebi et al., 2012 [28]	Laparoscopic repair (N-suture)	0.66 ± 0.18	0.68 ± 0.13	0.64 ± 0.10	0.309	NR	0.692
	Laparoscopic repair (Purse string)	0.66 ± 0.13	0.62 ± 0.09	0.64 ± 0.12	0.452	NR	0.673
	Open approach (High ligation)	0.59 ± 0.13	0.64 ± 0.09	0.60 ± 0.05	0.69	NR	0.337
Oral et al., 2019 [30]	Laparoscopic repair (PIRS)	0.66 ± 0.45	NR	0.60 ± 0.04	NR	0.175	NR
	Open approach (High ligation)	0.66 ± 0.07	NR	0.58 ± 0.04	NR	0.447	NR
Palabiyik et al., 2009 [25]	Open approach (High ligation)	0.56 ± 0.09	0.61 ± 0.14	0.57 ± 0.13	0.008 *	0.764	0.036 *
Basha et al., 2020 [27]	Open approach (High ligation)	0.67 ± 0.10	0.76 ± 0.10	0.68 ± 0.11	<0.0001 *	0.181	NR

Early follow-up: 1 day–1 week; Late follow-up: 1 month–6 months. Abbreviations: Preop, preoperative; postop, postoperative; RI, resistive index of the centripetal artery; FU, follow-up; SD, standard deviation; PIRS, percutaneous internal ring suture; NR, not reported. * indicates a statistically significant difference (*p* < 0.05).

**Table 3:** *p*-Values for comparison of means of resistive index of the centripetal artery after inguinal hernia repair with different surgical techniques, as reported by Çelebi et al. (2012) [28].

	Preoperative RI (p-Value)	Early Follow-Up RI (p-Value)	Late Follow-Up RI (p-Value)
N-suture vs. open	0.125	0.231	0.099
Purse string vs. open	0.063	0.436	0.140

Early follow-up: 1 week–1 month; late follow-up: 1 month–6 months. Abbreviations: RI, resistive index of the centripetal artery.

**Table 4:** Testicular volume or the ratio of the testicular volume before and after inguinal hernia repair in the respective study populations.

Study	Technique	Preop TV (Mean + SD)	Early Postop FU TV (Mean + SD)	Late Postop FU TV (Mean + SD)	Preop vs. Early FU (*p*-Value)	Preop vs. Late FU (*p*-Value)	Ratio TV Preop	Ratio TV Postop	Preop vs. Postop (*p*-Value)
Basha et al., 2020 [27]	Open approach (high ligation)	0.779 ± 0.395	0.741 ± 0.226	0.780 ± 0.310	0.25	0.98	NR	NR	NR
Parelkar et al., 2011 [26]	Laparoscopic repair (Purse string)	1.22 ± 0.52	NR	1.23 ± 0.54	NR	0.291	1.029 ± 0.103	1.022 ± 0.092	0.161
Leung et al., 1999 [34]	Open approach (high ligation)	NR	NR	NR	NR	NR	NR	1.023 95% CI [0.988–1.058]	NR

Early follow-up: 1 week–1 month; late follow-up: 1 month–6 months. Ratio = (testicular volume of operated side)/(testicular volume of healthy side). Abbreviations: Preop, preoperative; postop, postoperative; TV, testicular volume; FU, follow-up; SD, standard deviation; NR, not reported; CI, confidence interval.

## Data Availability

No new data were created or analyzed in this study. Data sharing is not applicable to this article. All data presented in this article are available at the above-mentioned databases (PubMed/Medline, Embase, Cochrane Library and Web of Science).
